# Expression of the Murine Norovirus (MNV) ORF1 Polyprotein Is Sufficient to Induce Apoptosis in a Virus-Free Cell Model

**DOI:** 10.1371/journal.pone.0090679

**Published:** 2014-03-05

**Authors:** Morgan R. Herod, Omar Salim, Rachel J. Skilton, Cynthia A. Prince, Vernon K. Ward, Paul R. Lambden, Ian N. Clarke

**Affiliations:** 1 Molecular Microbiology Group, University of Southampton, Southampton, United Kingdom; 2 Otago School of Medical Sciences, Department of Microbiology and Immunology, University of Otago, Dunedin, New Zealand; Wuhan Bioengineering Institute, China

## Abstract

Investigations into human norovirus infection, replication and pathogenesis, as well as the development of potential antiviral agents, have been restricted by the lack of a cell culture system for human norovirus. To date, the optimal cell culture surrogate virus model for studying human norovirus biology is the murine norovirus (MNV). In this report we generate a tetracycline-regulated, inducible eukaryotic cell system expressing the entire MNV ORF1 polyprotein. Once induced, the MNV ORF1 polyprotein was faithfully processed to the six mature non-structural proteins that predominately located to a discrete perinuclear region, as has been observed in active MNV infection. Furthermore, we found that expression of the ORF1 polyprotein alone was sufficient to induce apoptosis, characterised by caspase-9 activation and survivin down-regulation. This cell line provides a valuable new tool for studying MNV ORF1 non-structural protein function, screening for potential antiviral agents and acts as a proof-of-principle for such systems to be developed for human noroviruses.

## Introduction

Human norovirus infection is the leading cause of acute non-bacterial gastroenteritis, with epidemics common in semi-enclosed communities such as hospitals, schools and cruise ships. Currently, there is no vaccine or anti-viral treatment for norovirus infection.

The lack of an efficient cell culture system for studying human noroviruses [1] has limited studies on their infection, replication and pathogenesis as well as the development of potential antiviral treatments. Attempts to establish a human norovirus replicon system have had limited application since the system was first described in 2006 [2]–[4]. Most studies of human norovirus molecular biology have focused on using purified proteins and *in vitro* systems which are far removed from the cellular environment of viral infection [5]–[14].

Noroviruses belong to a genus within the calicivirus family of single stranded positive-sense RNA viruses. The norovirus genome is translated into three open reading frames (ORFs), termed ORF1, ORF2 and ORF3 [15], [16]. ORF1 encodes a single polyprotein which is proteolytically cleaved into six viral non-structural (NS) proteins (NS1/2, NS3, NS4, NS5, NS6 and NS7) [10], [17]–[25], whereas ORF2 and ORF3 encode the two viral structural proteins, VP1 and VP2, respectively [26], [27]. The ORF1 polyprotein encodes the viral NS6 protease which is responsible for processing of the polyprotein at all five NS boundaries [17], [18], [25] which follows a preferred temporal order [17], [28]. Studies of ORF1 polyprotein processing in human noroviruses primarily use *in vitro* models with heterologously expressed protein or peptide substrate.

**Figure 1:**
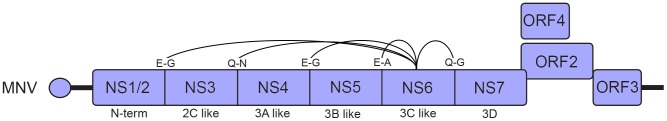
Schematic representation of the MNV genome. The genome of murine norovirus 1 is shown annotated with the five dipeptide ORF1 polyprotein cleavage sites that are cleaved by the viral NS6 protease. The ORF1 polyprotein is cleaved into the six NS proteins termed NS1/2, NS3, NS4, NS5, NS6 and NS7. Also indicated are the alternative names for each non-structural protein, N-term though to 3D.

The majority of our knowledge on human norovirus biology is drawn from studies with animal caliciviruses such as murine norovirus (MNV). The MNV genome bears a high degree of structural similarity to that of human norovirus, with the three main ORFs encoded within the human norovirus genome having a direct homologue in MNV (Figure 1). Additionally, the presence of a 4^th^ open reading frame, ORF4, has been indentified in MNV, which seems to have no direct homologue in human noroviruses [29], even though the presence of a non-equivalent 4^th^ open reading frame has been postulated within the genome of some strains [30]. The pathogenesis of MNV infection is markedly different from human noroviruses: human norovirus infection typically results in acute gastroenteritis, whereas MNV infects hematopoietic cells and is typically asymptomatic in immune-competent mice [31], [32]. Furthermore, *in cellulo* studies using MNV infection also suffer from unco-ordinated expression and a relatively short time window in which replication studies can be conducted before cells undergo apoptosis [33]. Attempts to establish MNV replicon systems have met with partial success, only transient expression of genome replication has been demonstrated and no permanently transformed cell line has been established [12].

Prior to the isolation of efficient cell culture models the study of other non-culturable pathogens such as hepatitis C virus, was advanced by the development of inducible eukaryotic cell models expressing full-length polyprotein or polyprotein precursors. Such models were subsequently used to study fundamental aspects of hepatitis C virus biology and provided a useful tool in the development of novel antiviral strategies in a well-defined and reproducible cellular context [34], [35].

Our aim was to establish an inducible eukaryotic cell system that would allow detailed studies of norovirus polyprotein processing without the need for infectious virus. As a proof-of-principle, we introduced the MNV ORF1 polyprotein under control of a tetracycline-regulated CMV promoter into HEK293 cells. We chose this approach since we could validate the system by comparison with MNV infected cells. Following induction, the cell clone, termed R1, expresses the full-length ORF1 polyprotein which is proteolytically cleaved into the six mature viral NS proteins. Immunofluorescence studies demonstrated NS1/2 through to NS5 proteins were found in a cellular localisation similar to that previously observed with MNV infected RAW246.7 cells [36], [37]. Approximately 16–24 hours post-induction cells underwent apoptosis which was characterised by the activation of caspase-9 and down-regulation of survivin, as has been previously described for wildtype MNV infection [33]. This inducible model of norovirus NS protein biosynthesis will provide a valuable tool for studying MNV ORF1 polyprotein processing and NS protein function in eukaryotic cells in the absence of viral infection and provides proof-of-principle for authentic expression and processing of norovirus NS proteins.

## Materials and Methods

### Plasmid Constructs

The tetracycline-inducible system for MNV ORF1 was constructed using the T-REx tetracycline-regulated expression system using component plasmid pcDNA6/TR (Invitrogen) with plasmid pcDNA4/TO/MNV ORF1.

The tetracycline-regulated CMV driven polII expression construct pcDNA4/TO/MNV ORF1 was derived from the T-REx component plasmid pcDNA4/TO using standard molecular cloning techniques in a three step process. Firstly, the C-terminal NS7 region of the MNV ORF1 polyprotein was amplified by PCR using primers 5′-cccgtgcttttggccctttctgt and 5′-ccccccgggccctcactcatcctcattcacaaag with template pMNV* [38] until the 3′ end of ORF1 and incorporating an unique *Apa*I site. The *Xho*I-*Apa*I digested PCR fragment was introduced into *Xho*I-*Apa*I digested pcDNA4/TO (Invitrogen) to make pcDNA4/TO/MNV/S1 (Figure S1). Subsequently, the 3596 bp *Eco*RV-*Xho*I fragment from pMNV*, representing the majority of ORF1 from the start of NS3 until the *Xho*I site in NS7, was cloned into *Eco*RV-*Xho*I digested pcDNA4/TO/MNV/S1 to create pcDNA4/TO/MNV/S2 (Figure S2). Finally, PCR was used with primer pair 5′-cccccctccggagtgaaatgaggatggcaacg and 5′-agagccgagttggtggaagc with pMNV* template, to amplify the N-terminal region of the ORF1 polyprotein from the 5′ end of MNV with flanking *Bsp*EI site until the *Eco*RV site. The PCR fragment was digested with *Bsp*EI and *Eco*RV and cloned into *Bsp*EI-*Eco*RV digested pcDNA4/TO/MNV/S2 to create pcDNA4/TO/MNV ORF1 (Figure S3 and S4).

### Cell Culture, Transfection and Infection

HEK293 cells (obtained from ATCC) were grown in DMEM supplemented with 10% foetal calf serum and GlutaMAX-1 (Invitrogen).

Stable cell lines were produced using the T-REx tetracycline-regulated expression system for mammalian cells (Invitrogen). Transfection of HEK293 cells was performed using FuGENE HD (Promega) using 2 µg of plasmid DNA and 5 µl of transfection reagent per well. For selection of stable cell lines, transfected cells were allowed to recover for 48 hours before selection with full growth medium supplemented with 5 µg/ml blasticidin (Invitrogen) and/or 200 µg/ml zeocin (Invitrogen). For infections, 10^6^ mouse macrophage RAW264.7 cells were seeded per well in a 12 well tray. Once the cells were attached, the cells were infected with MNV-CW1 at an MOI = 1. Cells were incubated with the virus at 37°C for 90 minutes and then the media changed. Time points were taken at 4, 8, 16, and 24 hours by scraping up the cells and then processing with NucleoSpin RNA/Protein extraction kit (Macherey-Nagel). Protein in a final volume of 100 µl was produced, the protein concentration quantified by BSA assay and equal quantities loaded onto 4–20% SDS-PAGE gels for analysis.

### SDS-PAGE and Western Blot

SDS-PAGE and Western blots were carried out as described previously [38]. Primary antibodies used were rabbit polyclonals: anti-NS1/2, anti-NS4, anti-NS5, anti-NS6 (all generated by Prof Vernon Ward, University of Otago, using *E.coli* generated, purified, whole NS protein antigens) and mouse anti-NS3 and anti-NS7 monoclonals, (generated on our behalf by AbMART, Shanghai, using NS protein-specific peptides). Proform and cleaved caspase-9 was detected using an anti-caspase-9 mouse monoclonal antibody, clone 96-2-22 (Merck Millipore). Survivin was detected using an anti-survivin rabbit monoclonal antibody, clone 71G4B7 (Cell Signalling Technology). Rabbit polyclonal anti-GAPDH antibody (ab9485- Abcam) was used to demonstrate gel loading, this antibody was used to probe both HEK293 cells and MNV infected RAW264.7 cells, in the murine RAW264.7 cells it had a high background. Goat anti-rabbit and goat anti-mouse HRP conjugates (Sigma-Aldrich) were employed as secondary reagents.

### Confocal Microscopy

R1 cells seeded onto polylysine coated glass coverslips were induced with 0.25 µg/ml tetracycline for 1 hour or left uninduced, fixed at timepoints between 2 and 24 hours post-induction in 4% paraformaldehyde, washed in PBS and permeabilised in saponin buffer (0.1% saponin, 10% foetal calf serum, 0.1% sodium azide) for 1 hour at 4°C. Primary and secondary antibodies were incubated in saponin buffer for 1 hour at room temperature and washed in saponin buffer between steps. Primary antibodies used were as described in western blotting (above) and were detected with anti-rabbit-Alexa488 or anti-mouse-Alexa488 conjugate (Invitrogen) secondaries. Cells were counterstained with 1 µg/ml DAPI (Fisher Scientific), washed a final time in PBS and mounted onto slides with ProLongGold (Invitrogen). Images were captured using a Leica TCP SP5 confocal microscope.

### Fluorescent Apoptosis Staining

To differentiate between apoptotic and non-apoptotic pathways, cells were stained with the Vybrant Apoptosis Assay (Invitrogen), following manufacturer’s protocol with slight modification. R1 cells seeded onto polylysine coated glass coverslips were induced with 0.25 µg/ml tetracycline (Sigma-Aldrich) for 1 hour or left uninduced and at timepoints between 12 and 24 hours post-induction stained with YO-PRO-1 and propidium iodide diluted in full growth medium, on ice for 30 minutes. Coverslips were mounted onto slides using PBS and imaged immediately using a Leica Leitz-DMRB fluorescent microscope with camera.

## Results

### A Stable Cell Clone Expressing MNV ORF1

**Figure 2:**
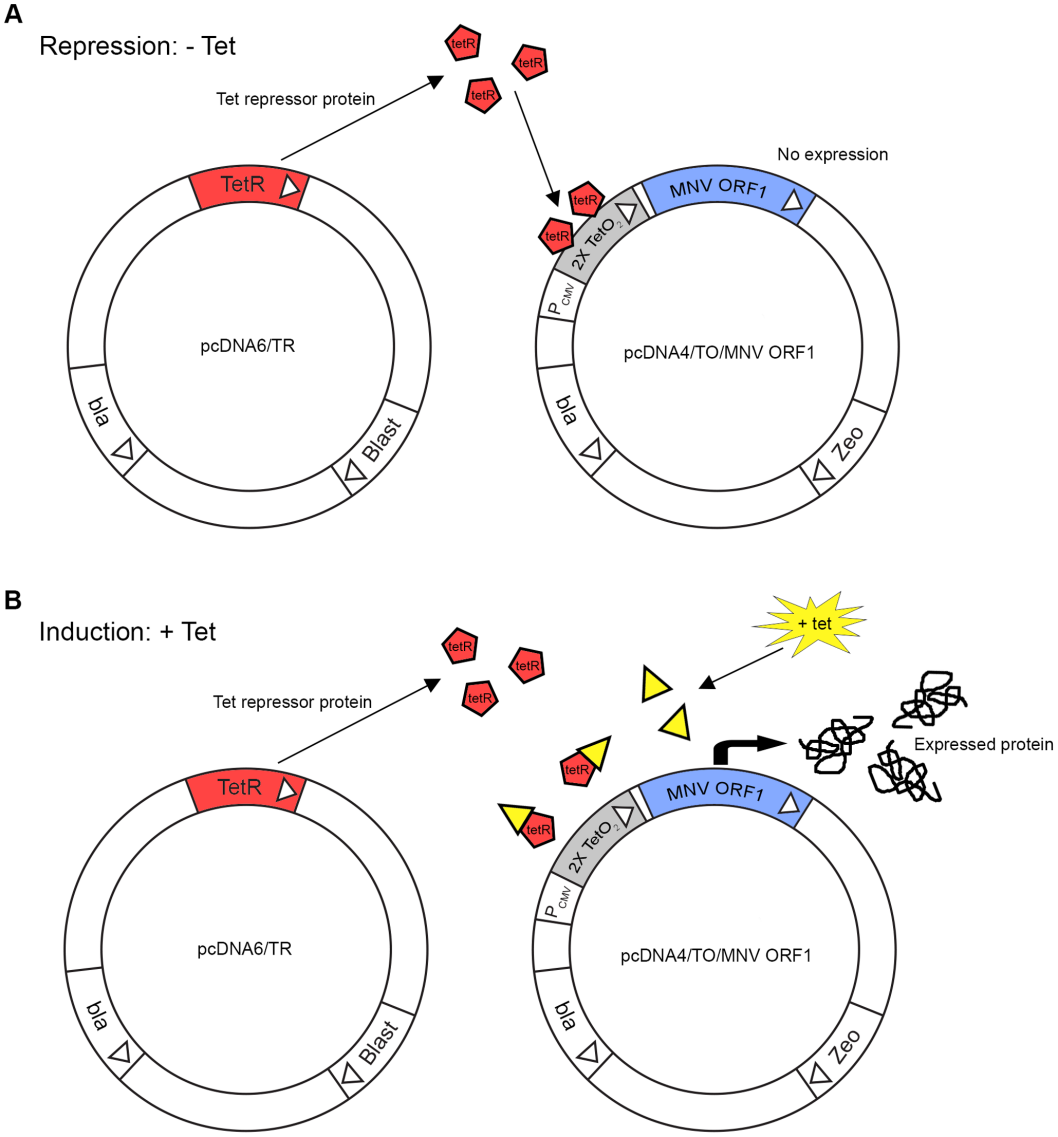
Schematic representation of the tetracycline-regulated ORF1 expression system. In the absence of tetracycline (Tet) (**A**) Tet repressor protein (TetR) homodimers, expressed constitutively from pcDNA6/TR, bind to the Tet operator 2 (TetO_2_) sequences in the inducible ORF1 expression vector (pcDNA4/TO/MNV ORF1) to repress transcription of the MNV ORF1 polyprotein. Upon the addition of Tet (**B**) Tet binds to TetR homodimers, resulting in a conformational change, releasing it from the TetO_2_ sequences allowing expression of the MNV ORF1 polyprotein.

To establish a virus-free model for studying MNV ORF1 processing we utilised the commercial TREx tetracycline (Tet) -regulated dual plasmid expression system. This system uses the pcDNA6/TR regulatory plasmid which constitutively expresses the Tet repressor gene to repress expression from the expression plasmid, pcDNA4/TO, which was engineered to express the MNV ORF1 polyprotein (Figure 2). In the absence of tetracycline, Tet repressor homodimers bind to the Tet operator sequences present in the vector to repress MNV ORF1 expression. Upon addition, Tet binds to repressor homodimers, releasing the repressor from the operator sequences and de-repressing expression of MNV ORF1.

Set up of this system first required selection of a cell line stably expressing the Tet repressor, with the requirement that the cell line chosen must also support MNV replication. Attempts to select a stable RAW264.7 or BHK-21 cell line expressing the Tet repressor were unsuccessful after multiple attempts (data not shown). However, HEK293 clones which expressed the Tet repressor were readily selectable after transfection with pcDNA6/TR and culture in the presence of blasticidin for 2 weeks. Following clonal expansion in the presence of blasticidin, the repressor cell line stably expressing the Tet repressor was transfected with the Tet responsive MNV ORF1 expressing plasmid, pcDNA4/TO/MNV ORF1. Transformants were selected for two weeks in the presence of blasticidin and zeocin and clonally expanded for a further 4 weeks. Six individual stable clones were screened for MNV ORF1 translation products by Western blot and a single clone, termed R1, was selected based on high level expression of the NS1/2 and NS7 proteins (data not shown).

### The ORF1 Polyprotein is Processed to Completion in the Absence of Viral Genomic RNA

**Figure 3:**
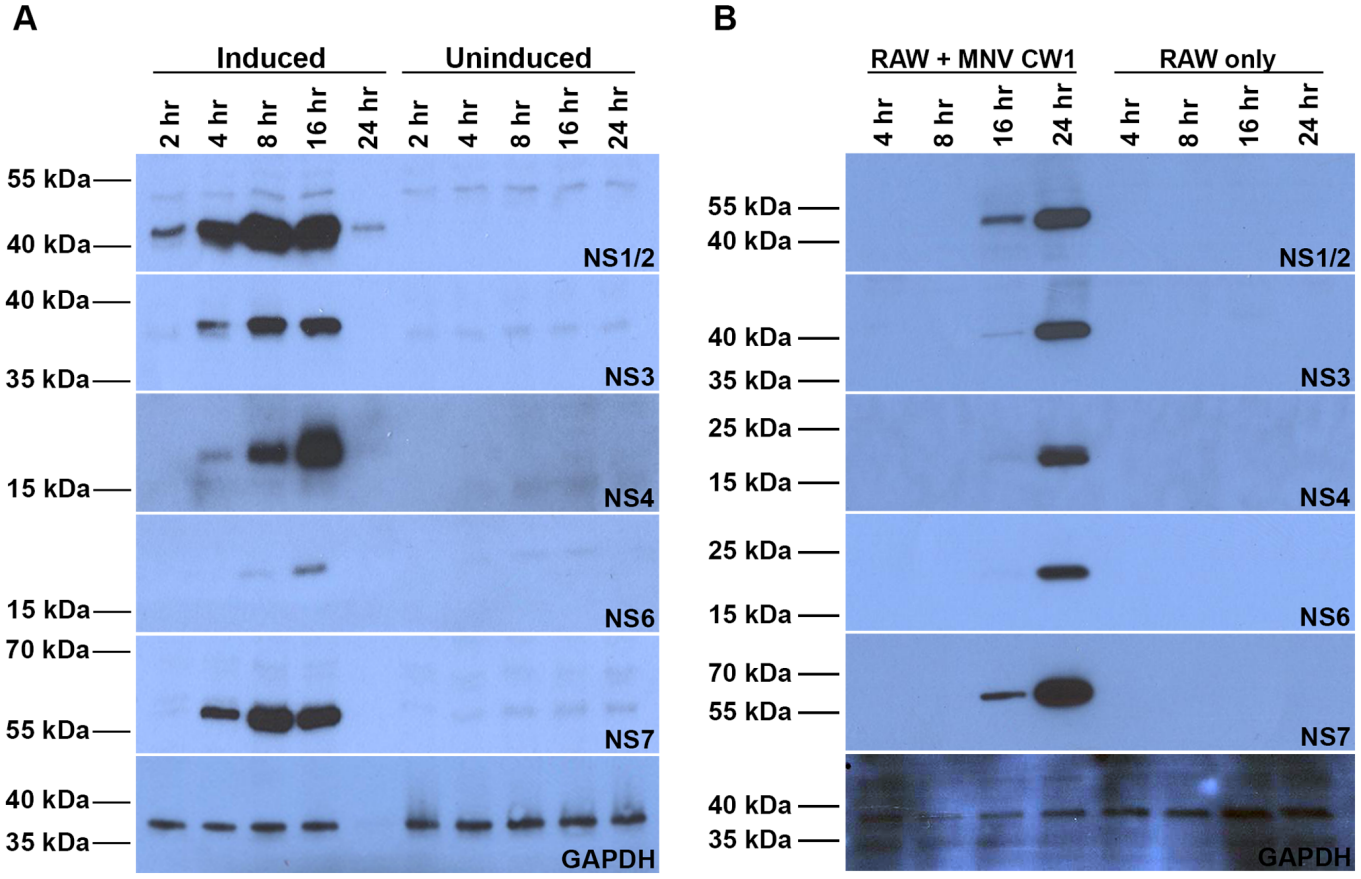
Western blot analysis of NS protein expression in the R1 cell clone and in MNV-1 infected RAW264.7 cells. A. The R1 cell line was induced with 0.25 µg/ml tetracycline or uninduced. B. RAW264.7 cells were infected with MNV with an MOI = 1.0. In both cases cells were harvested at 2, 4, 8, 16 and 24 hours post-induction/post infection, total protein quantified by BCA assay and 10 µg of each lysate analysed by Western blotting for expression of MNV NS proteins as indicated. In panel A, lysates were analysed by single concentration SDS PAGE. In panel B, 4–20% gradient SDS-PAGE was used, accounting for variations in relative migration of some of the molecular weight markers to those in panel A.

In MNV infection the ORF1 polyprotein is processed to completion by the NS6 protease, yielding the mature viral NS proteins in equal amounts. To establish whether processing of the ORF1 polyprotein goes to completion in the R1 cell clone, induced and uninduced cells were harvested at time points of 2, 4, 8, 16 and 24 hours post-induction and lysates probed by Western blot using antibodies against MNV NS proteins (Figure 3A). For comparison an equivalent time course was conducted with MNV infected cells and NS protein expression probed by Western blot (Figure 3B). Expression of most, fully cleaved, mature NS proteins could clearly be detected in the R1 cell line by 16 hours post-induction. In an active MNV infection expression of NS1/2, NS3, NS4 and NS7 could be easily detected from 16 hours post-infection while NS6 was only abundant at 24 hours post-infection. Furthermore, the molecular weight of all fully cleaved NS proteins expressed in the R1 cell line corresponded to the molecular weight as observed in MNV infected RAW264.7 cells. No significant amounts of uncleaved precursors could be detected with these antibodies within the R1 cell line or MNV infection at any time points even after over development except for NS5 (data not shown). Interestingly, between 22–24 hours post-induction, R1 cells underwent synchronised cell death to become detached from the surface, accounting for the lower protein concentration and the apparent reduced protein expression at the 24 hour time point.

### Cellular localisation of ORF1 Proteins

**Figure 4:**
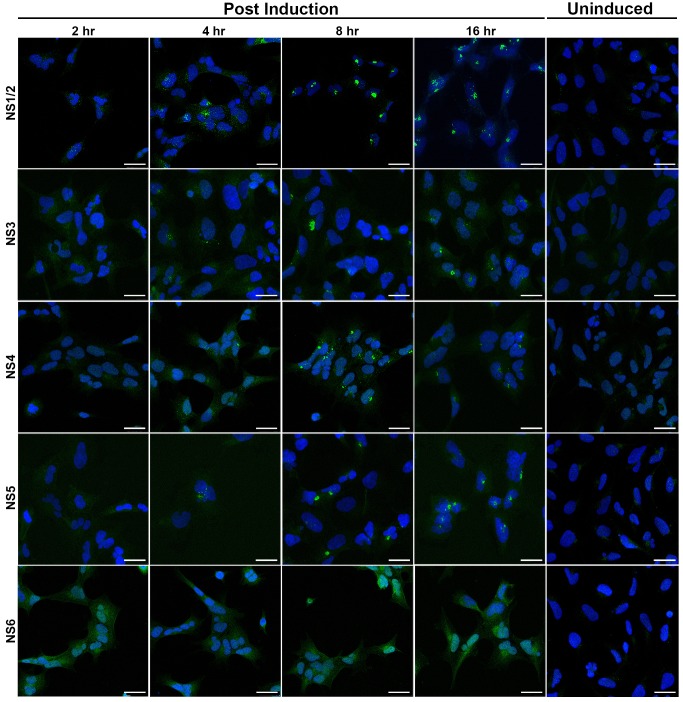
Localisation of MNV NS proteins when expressed in HEK293 cells. R1 cells were induced with 0.25 µg/ml tetracycline and fixed at 2, 4, 8, 16 and 24 hours post-induction before being labelled with antisera specific to MNV NS proteins (green) and cell nuclei were counterstained (blue). Images were captured by confocal microscopy. Scale bar is 25 µm.

Upon induction with tetracycline, the R1 clone demonstrated complete processing of the ORF1 polyprotein with mature NS proteins clearly detectable by 16 hours post-induction. Previous studies have defined the cellular localisation of MNV NS proteins expressed both individually by transfection of cDNA and in active MNV infection [36], [37], [39]. To determine the localisation of NS proteins in the R1 cell clone, induced and uninduced R1 cells on coverslips were fixed at equal time points 2–20 hours post-induction. The coverslips were individually labelled with antibodies against NS proteins and analysed by immunofluorescence (Figure 4). By 8 hours post-induction, NS1/2, NS3, NS4 and NS5 all localised to a discrete perinuclear region with little diffuse cytoplasmic staining. In contrast, NS6 demonstrated diffuse cytoplasmic staining at all time points with no defined perinuclear localisation apparent. Our antisera to NS7 did not work in immunofluoresecence.

### Expression of MNV ORF1 is Sufficient to Induce Apoptosis

**Figure 5:**
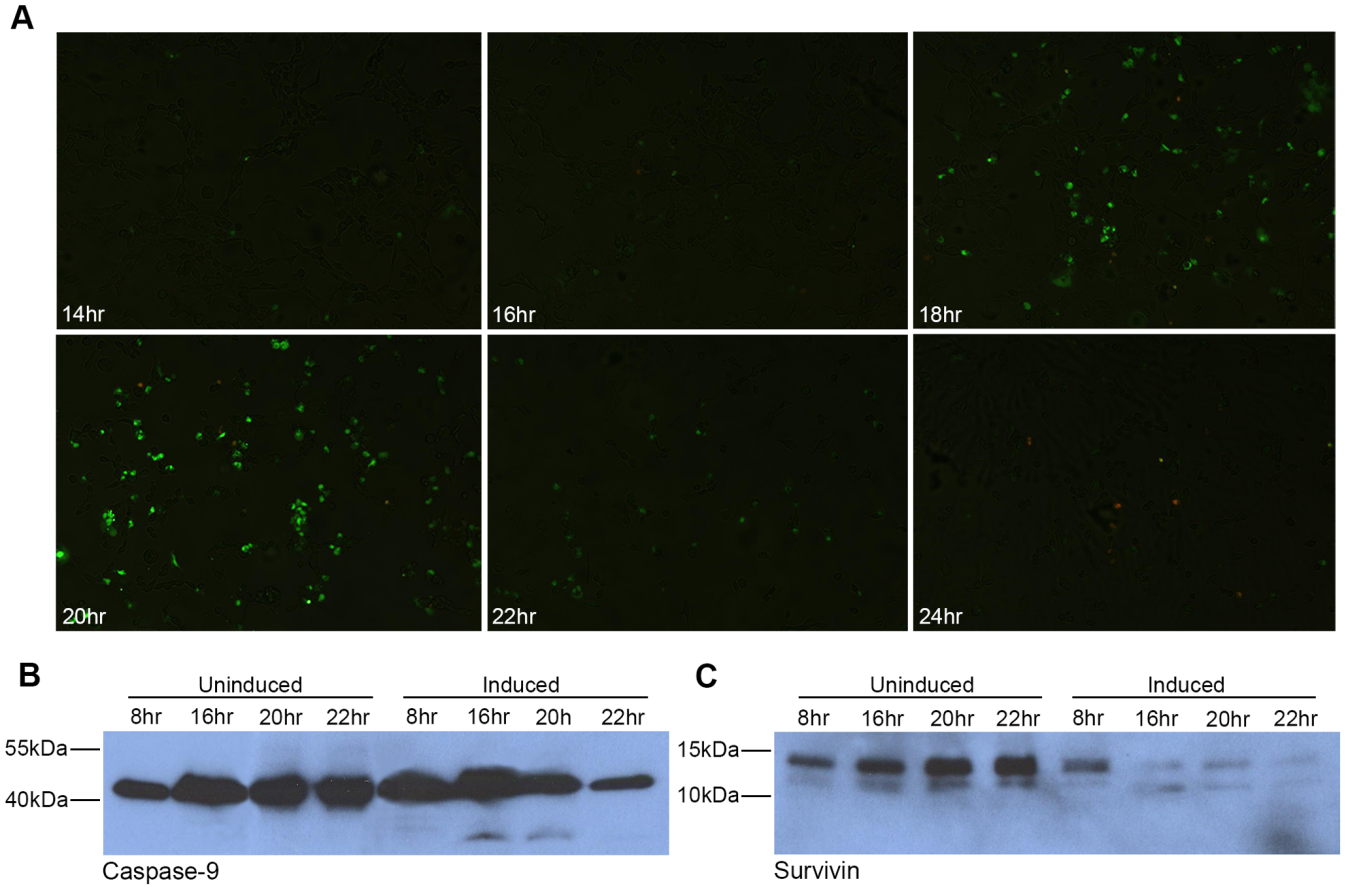
Expression of the MNV ORF1 polyprotein is sufficient to induce apoptosis in HEK293 cells. (A) R1 cells were seeded onto glass coverslips, induced with tetracycline and at 14, 16, 18, 20, 22 and 24 hours post-induction, stained with YO-PRO-1 (green) and propidium iodide (orange) and visualised by fluorescence microscopy. Western blot analysis of induced or uninduced R1 cells harvested at 4, 8, 16 and 24 hours post-induction for caspase-9 activation (B) or at 8, 16, 20 and 22 hours for survivin expression (C). For each lysate total protein quantification was performed by BCA assay and 50 µg of protein loaded per lane. Due to low protein concentration in the induced 24 hour sample approximately 100-fold less protein was loaded.

The R1 cells underwent synchronised cell death approximately 20–24 hours post-induction. MNV infection of RAW264.7 cells results in apoptosis beginning at 12–16 hours post-infection and eventually leading to cell death approximately 24 hours post-infection. MNV induced apoptosis is associated with activation of caspase-9, caspase-3, down-regulation of survivin and apoptosis through the mitochondrial pathway [33]. To determine whether the same events were responsible for the cell death observed with induced R1 cells it was first necessary to confirm the R1 cell death was attributed to apoptosis. Induced and uninduced R1 cells were therefore stained with the Vybrant Apoptosis Assay kit at time points between 12–24 hours post-induction and visualised by immunofluorescence (Figure 5A). With this system cells undergoing apoptosis exhibit bright green fluorescence and necrotic cells are labelled orange. Upon induction R1 cells underwent partially synchronised apoptosis, beginning at 16–18 hours post-induction and peaking at 20 hours post-induction. At times beyond 22 hours post-induction most cells had died and become detached from the surface.

To identify whether the observed apoptosis was associated with caspase-9 activation and survivin down-regulation (as has been observed with MNV infection [33]), induced or uninduced R1 cells were harvested at timepoints between 8 and 22 hours post-induction and lysates probed by Western blot using anti-caspase-9 and anti-survivin monoclonal antibodies (Figure 5B and 5C). Caspase-9 activation could only be detected in induced R1 cells, at 16 hours post-induction as indicated by the cleaved caspase-9 product of approximately 37 kDa by Western blot. By 22 hours post-induction most of the cells had detached from the surface, therefore resulting in a lower protein concentration in this sample and reduced protein loading. In MNV infected RAW264.7 cells survivin down-regulation begins at approximately 8 hours post-infection [33]. Concordant with this study, down-regulation of survivin can clearly be observed in induced R1 cells at 8 hours post-induction and is maintained down-regulated up to 22 hours post induction.

## Discussion

Our aim was to construct a human cell line capable of inducible expression of norovirus non-structural proteins (ORF1). We describe a stable cell line expressing the MNV ORF1 polyprotein under control of a tightly-regulated inducible tetracycline gene expression system. At the start of this study, attempts to select stable transformants were performed with three well characterised cell lines, RAW264.7, BHK-21 and HEK293. These cell lines were chosen based on their relatively high transfection efficiencies, and because it is possible to recover MNV from all three cell lines following transfection with full-length viral cDNA [38], [40]. Despite multiple attempts, we were unable to select stable cell lines with RAW264.7 and BHK-21 cells. In contrast, stable HEK293 transformants were readily selectable and an individual clone, termed R1, was chosen for characterisation based on high expression of the non-structural proteins at 5′ and 3′ end of the ORF1 polyprotein, NS1/2 and NS7, respectively.

Upon induction, the MNV ORF1 polyprotein was processed to completion, similar to the processing seen in MNV infection [25]. Expression of the N-terminal ORF1 protein, NS1/2, was just detectable after only 2 hours post-induction, closely followed by NS3, NS4 and NS7 detectable after 4 hours and NS6 at 8 hours post-induction, with expression peaking at approximately 8–16 hours. For each NS protein the time course of expression in R1 cells was faster than that observed in MNV infected RAW264.7 cells. However, there was little difference in the final relative amount of each NS protein expressed in the R1 cells and an active MNV infection run in parallel and using the same reagents for immune detection. The early, high level expression of the MNV ORF1 polyprotein from the CMV promoter in the R1 clone allows for greater initial synthesis of viral NS protein compared to a natural infection (where viral genome amplificiation has to occur before high-level protein expression is achieved) and as a result may contribute to the induction of apoptosis observed in R1 cells.

Mature cleavage products were readily detectable in both wild type MNV infection and the induced R1 clone. It is likely that complete processing of the ORF1 polyprotein is essential for viral RNA replication, making the protease an attractive target for anti-viral therapy. The apparent processing to completion of the ORF1 polyprotein in R1 cells clearly demonstrates that the NS6 protease retains activity in this model system. The R1 cell line will therefore provide a useful tool for evaluating potential protease inhibitors in a tightly regulated, reproducible, eukaryotic system that is free from viral replication where secondary factors such as unco-ordinated expression can have interfering effects.

In RAW264.7 cells all MNV NS proteins locate to a distinct perinuclear region along with dsRNA and it is thought to be the location of viral RNA replication [36], [37]. In our model system the first four NS proteins, NS1/2, NS3, NS4 and NS5, all located to a perinuclear region similar to that observed in MNV-infected RAW246.7 cells. In contrast, NS6 demonstrated largely diffuse, cytoplasmic fluorescence with no distinguishable perinuclear localisation.

Following induction of the ORF1 polyprotein R1 cells undergo cell death and become detached from the surface after approximately 24 hours, which was not observed in uninduced R1 cells or an inducible HEK293 cell line expressing β-galactosidase (data not shown). The observed ORF1 related cell death was characterised by induction of apoptosis at approximately 16 hours post-induction and associated with the activation of caspase-9 and down-regulation of survivin. These observations are in agreement with previous findings where it was shown that MNV replication triggered apoptosis in infected RAW264.7 cells at 12–16 hours post-infection, through the activation of caspase-9, caspase-3 and was associated with a down-regulation of survivin (an inhibitor of apoptosis which prevents activation of caspase-9). Furthermore, both UV inactivation of virus or treatment with cycloheximide eliminated apoptosis and the expression of individual structural or non-structural proteins was shown not to result in down-regulation of survivin, suggesting active viral replication was required to induce apoptosis [33]. Here we report that only expression of the MNV ORF1 polyprotein is sufficient to induce survivin down-regulation and apoptosis in the complete absence of viral genome or viral genome replication. Taking these data together, it seems likely that the survivin down-regulation and induction of apoptosis is mediated by one or more ORF1 polyprotein products, but not by a single NS protein expressed alone or by viral genome replication. However, it is unclear whether this may be the effect of several mature non-structural proteins working in co-operation and/or transient ORF1 polyprotein precursor products.

It is not known what role apoptosis and programmed cell death may play in MNV infection. In RAW264.7 cells MNV infection clearly causes apoptosis and programmed cell death, most likely through an atypical pathway involving survivin and possibly other players such as cathepsin B [33], [41]. Blocking apoptosis using a pan-caspase inhibitor both accelerated cell death and changed the death pathway to typical necrosis, while resulting in an over 10-fold reduction in infectious virion production [41]. It was therefore suggested MNV has adapted a strategy to allow apoptosis to proceed and thus provide a larger time window for virus replication. Alternatively, activation of caspases may provide a vital role in the viral replication process. The MNV ORF1 N-terminal protein, NS1/2, has been shown to be processed by caspase-3 into two fragments of 13.6 and 24.7 kDa [25], the first of which contains the majority of the disordered region [42]. The function of the NS1/2 protein is not known and it is likely to perform multiple roles during replication. However, it is possible that cleavage of the NS1/2 protein by activated caspase-3 may be a functional requirement of this protein.

In the absence of a human norovirus cell culture system most of our knowledge of norovirus molecular biology comes from studies with MNV. There is a human norovirus replicon system but this suffers from low level replication and an absence of mature ORF1 polyprotein cleavage products hindering certain approaches such as pulse chase analyses and the development of protease inhibitors. By contrast the inducible norovirus R1 cell line described here is ideal for such studies which require co-ordinated high level expression and is well suited for applications such as high throughput enzyme inhibitors testing. In addition, the well-defined cellular architecture of the parental HEK293 cells used to establish the R1 cell line makes them easier to use than MNV infected RAW264.7 cells which have a smaller cytoplasm and less well defined cellular features.

In conclusion, we have established and characterised a stable cell line with tightly regulated inducible expression of the MNV ORF1 polyprotein which is processed into mature non-structural proteins. This cell line will provide a valuable tool for studying polyprotein processing and non-structural protein function in eukaryotic cells, for screening for novel protease inhibitors and provides a proof-of-principle for similar systems to be established with non-culturable human noroviruses.

## Supporting Information

Figure S1 **Map of plasmid pcDNA4/TO/MNV/S1.** PCR was used to amplify the ORF1 C-terminal region from pMNV* from upstream of the *Xho*I site until the 3′ end and incorporating a downstream *Apa*I site. The *Xho*I-*Apa*I digested PCR fragment was ligated into *Xho*I-*Apa*I digested pcDNA4/TO at the multiple cloning site to create pcDNA4/TO/MNV/S1.
(TIF)

Figure S2 **Map of plasmid pcDNA4/TO/MNV/S2.** Plasmid pcDNA4/TO/MNV/S2 was created by ligating the *Eco*RV-*Xho*I fragment from pMNV*, representing the large central portion of the MNV ORF1 region, into *Eco*RV-*Xho*I digested pcDNA4/TO/MNV/S1 to give pcDNA4/TO/MNV/S2.
(TIF)

Figure S3 **Map of plasmid pcDNA4/TO/MNV ORF1.** Plasmid pcDNA4/TO/MNV ORF1 was created by PCR amplifying the ORF1 N-terminal region of pMNV* with flanking upstream *Bsp*EI site until the *Eco*RV site and ligating the *Bsp*EI-*Eco*RV PCR fragment into *Bsp*EI-*Eco*RV digested pcDNA4/TO/MNV/S2.
(TIF)

Figure S4 **Plasmid pcDNA4/TO/MNV ORF1 features table and sequence.** The main features of plasmid pcDNA4/TO/MNV ORF1 are shown with the nucleotide co-ordinates and the full plasmid nucleotide sequence.
(DOC)
