# Supplementary figures and images for: Expression of the Murine Norovirus (MNV) ORF1 Polyprotein Is Sufficient to Induce Apoptosis in a Virus-Free Cell Model
Source: PLoS One. 2014 Mar 5;9(3):e90679. doi: 10.1371/journal.pone.0090679 (PMC3944349; doi:10.1371/journal.pone.0090679)

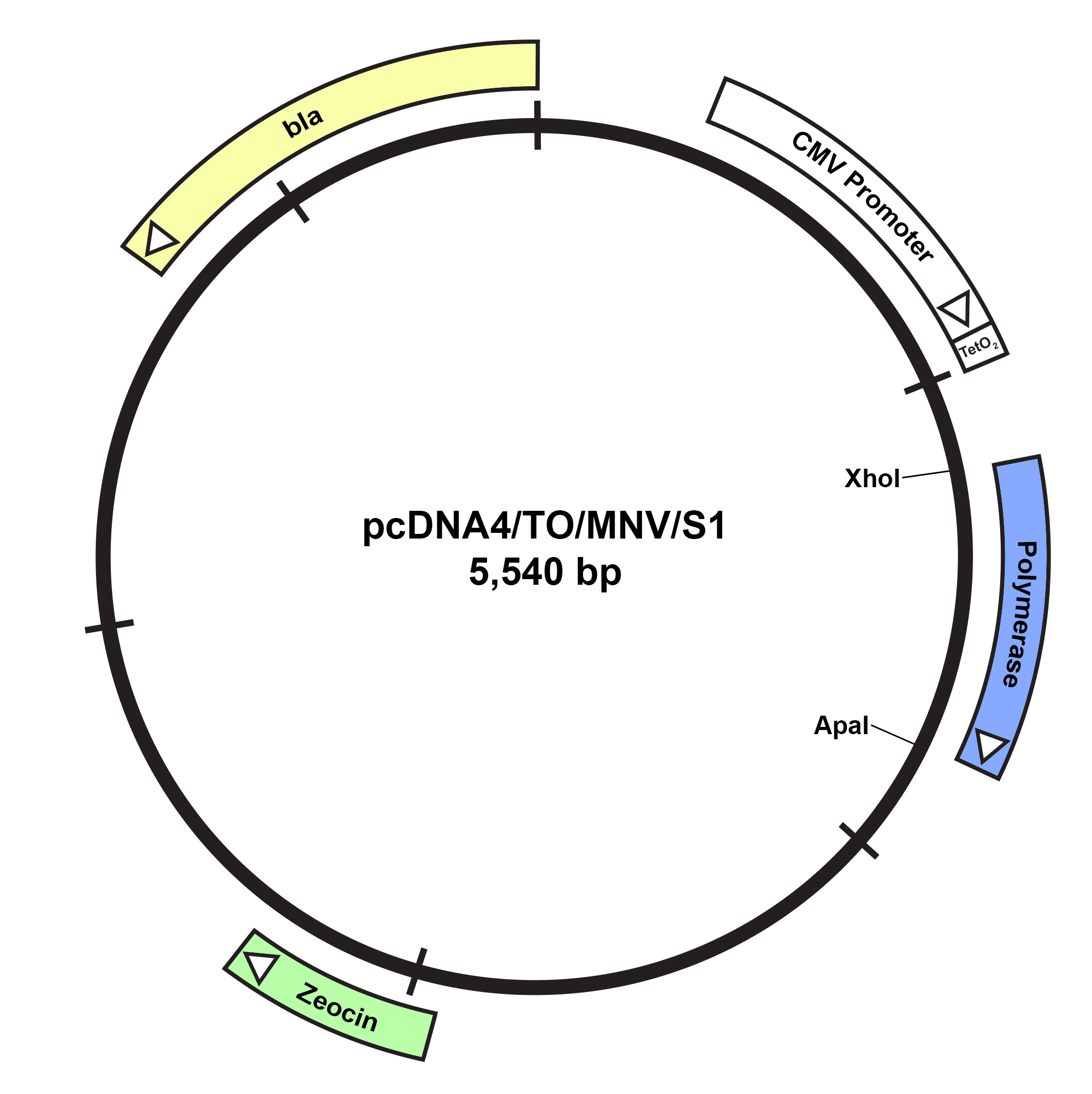

Supplement: Figure S1 — Map of plasmid pcDNA4/TO/MNV/S1. PCR was used to amplify the ORF1 C-terminal region from pMNV* from upstream of the XhoI site until the 3′ end and incorporating a downstream ApaI site. The XhoI-ApaI digested PCR fragment was ligated into XhoI-ApaI digested pcDNA4/TO at the multiple cloning site to create pcDNA4/TO/MNV/S1. (TIF) [file pone.0090679.s001.tif]

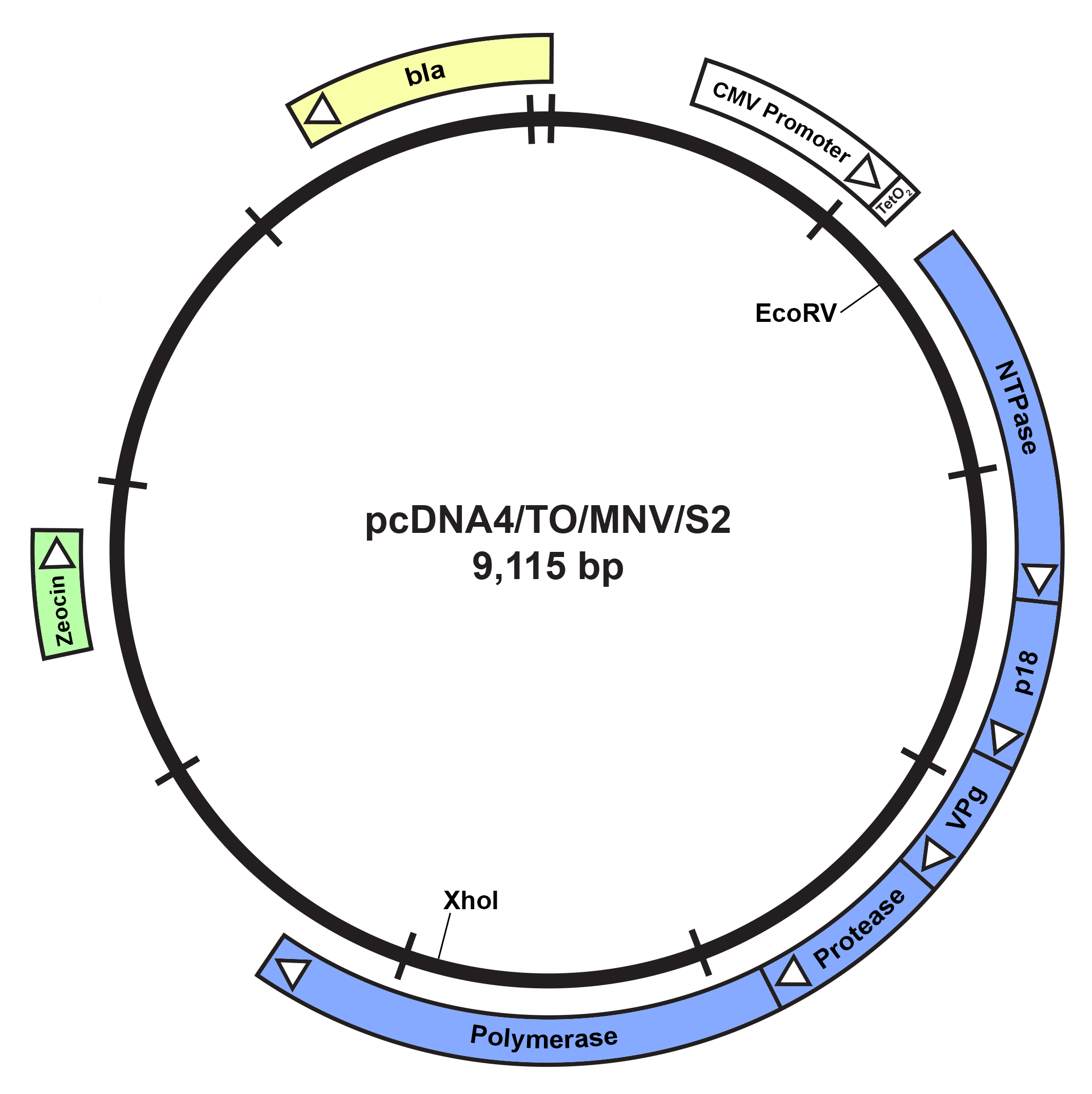

Supplement: Figure S2 — Map of plasmid pcDNA4/TO/MNV/S2. Plasmid pcDNA4/TO/MNV/S2 was created by ligating the EcoRV-XhoI fragment from pMNV*, representing the large central portion of the MNV ORF1 region, into EcoRV-XhoI digested pcDNA4/TO/MNV/S1 to give pcDNA4/TO/MNV/S2. (TIF) [file pone.0090679.s002.tif]

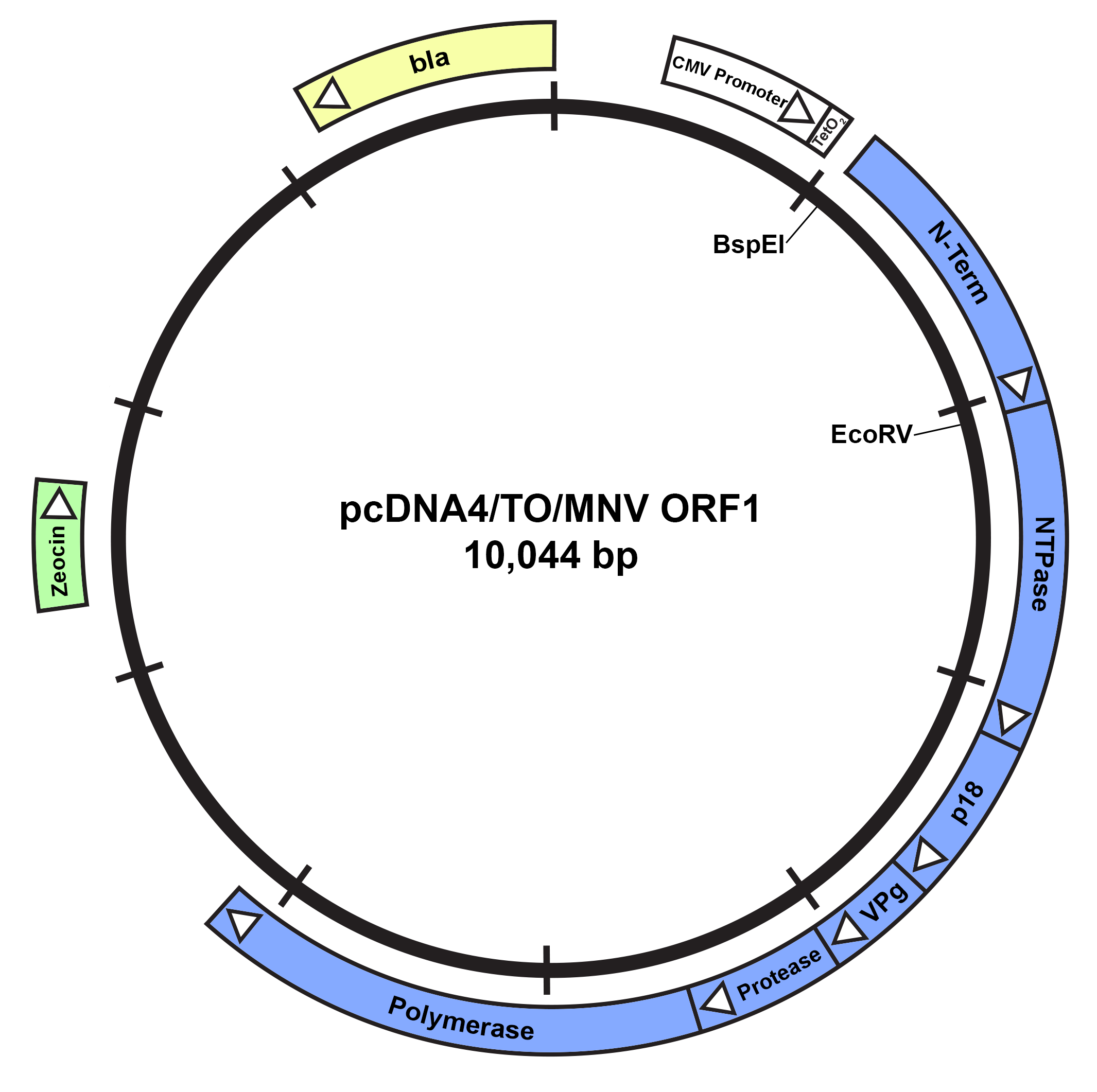

Supplement: Figure S3 — Map of plasmid pcDNA4/TO/MNV ORF1. Plasmid pcDNA4/TO/MNV ORF1 was created by PCR amplifying the ORF1 N-terminal region of pMNV* with flanking upstream BspEI site until the EcoRV site and ligating the BspEI-EcoRV PCR fragment into BspEI-EcoRV digested pcDNA4/TO/MNV/S2. (TIF) [file pone.0090679.s003.tif]
