# Supplementary material for: Expression of the Murine Norovirus (MNV) ORF1 Polyprotein Is Sufficient to Induce Apoptosis in a Virus-Free Cell Model
Source: PLoS One. 2014 Mar 5;9(3):e90679. doi: 10.1371/journal.pone.0090679 (PMC3944349; doi:10.1371/journal.pone.0090679)
Supplement: Figure S4 — Plasmid pcDNA4/TO/MNV ORF1 features table and sequence. The main features of plasmid pcDNA4/TO/MNV ORF1 are shown with the nucleotide co-ordinates and the full plasmid nucleotide sequence. (DOC) [file pone.0090679.s004.doc]

**Plasmid pcDNA4/TO/MNV ORF1 feature list**

| **Nucleotide position** | **Feature** | **Length** |
| --- | --- | --- |
| 232 - 958 | CMV promoter | 727 bp |
| 804 - 810 | TATA box | 7 bp |
| 820 - 859 | Tetracycline operator sequences | 40 bp |
| 961 - 6029 | MNV* sequence | 5069 bp |
| 6061 - 6285 | BGH poly(A) site | 225 bp |
| 7216 - 7590 | Zeocin resistance | 375 bp |
| 9908 - 9048 | β-lactamase | 861 bp |

**The sequence of pcDNA4/TO/MNV ORF1 (10,044 bp)**

**GACGGATCGG GAGATCTCCC GATCCCCTAT GGTGCACTCT CAGTACAATC TGCTCTGATG CCGCATAGTT AAGCCAGTAT CTGCTCCCTG CTTGTGTGTT GGAGGTCGCT GAGTAGTGCG CGAGCAAAAT TTAAGCTACA ACAAGGCAAG GCTTGACCGA CAATTGCATG AAGAATCTGC TTAGGGTTAG GCGTTTTGCG CTGCTTCGCG ATGTACGGGC CAGATATACG 230**

**CGTTGACATT GATTATTGAC TAGTTATTAA TAGTAATCAA TTACGGGGTC ATTAGTTCAT AGCCCATATA TGGAGTTCCG CGTTACATAA CTTACGGTAA ATGGCCCGCC TGGCTGACCG CCCAACGACC CCCGCCCATT GACGTCAATA ATGACGTATG TTCCCATAGT AACGCCAATA GGGACTTTCC ATTGACGTCA ATGGGTGGAG TATTTACGGT AAACTGCCCA 460**

**CTTGGCAGTA CATCAAGTGT ATCATATGCC AAGTACGCCC CCTATTGACG TCAATGACGG TAAATGGCCC GCCTGGCATT ATGCCCAGTA CATGACCTTA TGGGACTTTC CTACTTGGCA GTACATCTAC GTATTAGTCA TCGCTATTAC CATGGTGATG CGGTTTTGGC AGTACATCAA TGGGCGTGGA TAGCGGTTTG ACTCACGGGG ATTTCCAAGT CTCCACCCCA 690**

**TTGACGTCAA TGGGAGTTTG TTTTGGAACC AAAATCAACG GGACTTTCCA AAATGTCGTA ACAACTCCGC CCCATTGACG CAAATGGGCG GTAGGCGTGT ACGGTGGGAG GTCTATATAA GCAGAGCTCT CCCTATCAGT GATAGAGATC TCCCTATCAG TGATAGAGAT CGTCGACGAG CTCGTTTAGT GAACCGTCAG ATCGCCTGGA GACGCCATCC ACGCTGTTTT 920**

**GACCTCCATA GAAGACACCG GGACCGATCC AGCCTCCGGA GTGAAATGAG GATGGCAACG CCATCTTCTG CGCCCTCTGT GCGCAACACA GAGAAACGCA AAAACAAGAA GGCTTCGTCT AAAGCTAGTG TCTCCTTTGG AGCACCTAGC CCCCTCTCTT CGGAGAGCGA AGACGAAATT AATTACATGA CCCCTCCTGA GCAGGAAGCT CAGCCCGGCG CCCTTGCGGC 1150**

**CCTTCATGCG GAAGGGCCGC TTGCCGGGCT CCCCGTGACG CGTAGTGATG CACGCGTGCT GATCTTCAAT GAGTGGGAGG AGAGGAAGAA GTCTGATCCG TGGCTACGGC TGGACATGTC TGATAAGGCT ATCTTCCGCC GTTACCCCCA TCTGCGGCCT AAGGAGGATA GGCCTGACGC GCCCTCCCAT GCGGAGGACG CTATGGATGC CAAGGAGCCT GTGATCGGCT 1380**

**CTATCTTGGA GCAGGATGAT CACAAGTTTT ACCATTACTC TGTCTACATC GGTGGCGGCC TTGTGATGGG GGTCAACAAC CCCAGTGCTG CGGTCTGCCA GGCAACGATT GATGTGGAGA AGCTACACCT CTGGTGGCGG CCTGTCTGGG AGCCCCGCCA TCCCCTTGAC TCGGCTGAGT TGAGGAAGTG CGTGGGCATG ACTGTCCCCT ACGTGGCCAC CACCGTCAAC 1610**

**TGTTATCAGG TCTGCTGCTG GATTGTTGGC ATCAAGGACA CCTGGCTGAA GAGGGCGAAG ATCTCTAGAG ATCTGCCCTT CTACAGCCCC GTCCAGGACT GGAACGTCGA CCCCCAGGAG CCCTTCATTC CATCCAAGCT CAGGATGGTC TCGGATGGCA TCCTGGTGGC CTTGTCGGCA GTGATTGGCC GGCCAATTAA GAACCTACTG GCCTCAGTTA AGCCGCTCAA 1840**

**CATTCTCAAC ATCGTGCTGA GCTGTGATTG GACCTTTTCG GGCATTGTCA ATGCCCTGAT CTTGCTTGCT GAGCTCTTTG ACATCTTTTG GACCCCCCCT GATGTGACCA ACTGGATGAT ATCTATCTTC GGGGAATGGC AGGCCGAAGG GCCCTTCGAC CTTGCTCTTG ACGTGGTGCC CACCCTGTTG GGCGGGATCG GGATGGCTTT TGGCCTCACC TCTGAGACCA 2070**

**TCGGGCGCAA GCTCGCTTCC ACCAACTCGG CTCTCAAGGC CGCCCAAGAG ATGGGCAAGT TCGCCATAGA GGTCTTCAAG CAAATTATGG CCTGGATCTG GCCCTCTGAG GACCCAGTGC CAGCCCTCTT ATCCAACATG GAGCAGGCCA TCATTAAGAA TGAGTGTCAA CTGGAGAACC AACTCACGGC CATGTTGCGG GATCGCAACG CAGGGGCTGA ATTCCTAAGG 2300**

**TCCCTTGATG AGGAGGAGCA GGAAGTCCGC AAGATCGCAG CTAAGTGCGG CAACTCGGCC ACCACTGGAA CCACCAACGC TCTGCTGGCC AGGATCAGCA TGGCCCGCGC GGCCTTTGAG AAAGCTCGCG CTGAACAGAC CTCCCGAGTC CGCCCTGTGG TGATCATGGT CTCAGGCAGG CCCGGGATCG GGAAAACCTG CTTTTGCCAA AACCTGGCCA AGAGGATTGC 2530**

**TGCGTCCCTG GGTGATGAGA CCTCTGTTGG CATCATACCA CGCGCTGATG TCGACCACTG GGATGCTTAC AAGGGAGCCA GAGTGGTTCT CTGGGATGAT TTCGGCATGG ACAACGTGGT GAAGGATGCA CTGAGGCTTC AGATGCTTGC CGACACGTGC CCAGTGACAC TCAATTGTGA CAGGATTGAG AACAAGGGAA AGATGTTTGA CTCTCAGGTC ATTATCATCA 2760**

**CCACAAATCA ACAAACCCCC GTGCCCCTGG ACTATGTCAA CCTGGAGGCG GTCTGCCGCC GCATAGATTT CCTGGTTTAT GCTGAGAGCC CTGTTGTTGA TGATGCTCGG GCCAGAGCCC CTGGCGATGT GAATGCAGTG AAAGCTGCCA TGAGGCCCGA TTACAGCCAC ATCAATTTCA TCTTGGCACC GCAGGGCGGC TTTGACCGTC AGGGAAACAC CCCCTACGGT 2990**

**AAGGGCGTCA CCAAGATCAT TGGCGCCACT GCTCTTTGCG CGAGAGCGGT TGCTCTTGTC CATGAGCGCC ATGATGATTT CGGCCTCCAG AACAAGGTCT ATGACTTTGA TGCCGGCAAG ATCACCGCCT TCAAAGCCAT GGCGGCTGAC GCCGGCATTC CATGGTACAA AATGGCAGCT ATTGGGTGCA AAGCAATGGG GTGCACCTGT GTAGAGGAGG CCATGCATTT 3220**

**ACTTAAGGAT TATGAGGTGG CTCCCTGTCA GGTGATCTAC AATGGTGCCA CCTATAATGT GAGCTGCATC AAGGGTGCCC CAATGGTTGA AAAGGTCAAG GAGCCTGAAT TGCCCAAAAC ACTTGTCAAC TGTGTCAGAA GGATAAAGGA GGCCCGCCTC CGCTGCTACT GTAGGATGGC TGCTGACGTC ATCACGTCCA TTCTGCAGGC GGCCGGCACG GCCTTCTCTA 3450**

**TTTACCACCA GATTGAGAAG AGGTCTAGAC CATCCTTTTA TTGGGATCGT GGATACACCT ACCGTGACGG ACCTGGATCC TTTGACATCT TTGAGGATGA CGATGATGGG TGGTACCACT CTGAGGGAAA GAAGGGCAAG AACAAGAAGG GCCGGGGGCG ACCCGGAGTC TTCAGAACCC GTGGGCTCAC GGATGAGGAG TACGATGAAT TCAAGAAGCG CCGCGAGTCT 3680**

**AGGGGCGGCA AGTACTCCAT TGATGATTAC CTCGCTGACC GCGAGCGAGA AGAAGAACTC CTGGAGCGGG ACGAGGAGGA GGCTATCTTC GGGGATGGCT TCGGGTTGAA GGCCACCCGC CGTTCCCGCA AGGCAGAGAG AGCCAAACTG GGCCTGGTTT CTGGTGGCGA CATCCGCGCC CGCAAGCCGA TCGACTGGAA TGTGGTTGGC CCCTCCTGGG CTGACGATGA 3910**

**CCGCCAGGTC GACTACGGCG AGAAGATCAA CTTTGAGGCC CCAGTCTCCA TCTGGTCCCG TGTTGTGCAG TTCGGCACGG GGTGGGGCTT TTGGGTGAGC GGCCACGTCT TCATCACCGC CAAGCATGTG GCGCCCCCCA AGGGCACGGA GATCTTTGGG CGCAAGCCCG GGGACTTCAC TGTCACTTCC AGCGGGGACT TCTTGAAGTA CTACTTCACC AGCGCCGTCA 4140**

**GGCCTGACAT TCCCGCCATG GTCCTGGAGA ATGGGTGCCA GGAGGGCGTC GTCGCCTCGG TCCTTGTCAA GAGAGCCTCC GGCGAGATGC TTGCCCTGGC TGTCAGGATG GGTTCACAGG CCGCCATCAA GATTGGTAGT GCCGTTGTGC ATGGGCAAAC TGGCATGCTC CTGACTGGCT CTAATGCCAA GGCCCAGGAC CTCGGGACCA TCCCGGGCGA CTGTGGCTGT 4370**

**CCCTATGTTT ATAAGAAGGG TAACACCTGG GTTGTGATTG GGGTGCACGT GGCGGCCACT AGGTCTGGTA ACACAGTCAT TGCCGCCACT CACGGAGAAC CCACACTTGA GGCTCTGGAG TTCCAGGGAC CCCCCATGCT TCCCCGCCCC TCAGGCACCT ATGCAGGCCT CCCCATCGCC GATTACGGCG ACGCTCCCCC CTTGAGCACC AAGACCATGT TCTGGCGTAC 4600**

**CTCGCCAGAG AAGCTTCCCC CTGGGGCTTG GGAGCCAGCC TATCTCGGCT CTAAAGATGA GAGGGTGGAC GGTCCTTCCC TTCAGCAGGT CATGCGAGAT CAGCTTAAGC CCTATTCAGA ACCACGCGGT CTGCTTCCCC CTCAAGAAAT CCTTGATGCA GTCTGCGACG CCATTGAGAA CCGCCTTGAG AACACCCTTG AACCACAGAA GCCCTGGACA TTTAAGAAGG 4830**

**CTTGTGAGAG CTTGGACAAG AACACCAGTA GTGGGTATCC CTATCACAAG CAGAAGAGCA AGGACTGGAC GGGGAGCGCT TTTATTGGCG ATCTTGGTGA CCAGGCCACC CACGCCAACA ACATGTATGA GATGGGTAAA TCCATGCGAC CCATTTATAC AGCTGCCCTC AAGGATGAAC TGGTTAAGCC AGACAAGATC TACGGGAAGA TAAAGAAGAG GCTTCTCTGG 5060**

**GGCTCTGACC TTGGCACCAT GATTCGCGCT GCCCGTGCTT TTGGCCCTTT CTGTGATGCT CTGAAAGAAA CCTGCATTTT CAACCCCATC AGAGTGGGCA TGTCGATGAA CGAAGATGGC CCCTTCATCT TCGCAAGACA CGCCAATTTC AGGTACCACA TGGATGCTGA CTATACCAGG TGGGACTCCA CCCAACAGAG AGCCATCCTA AAGCGCGCTG GCGACATCAT 5290**

**GGTGCGCCTC TCCCCTGAGC CAGACTTGGC TCGGGTTGTC ATGGATGATC TCCTGGCCCC CTCGCTGTTG GACGTCGGCG ACTATAAGAT CGTTGTCGAG GAGGGGCTCC CATCCGGCTG CCCTTGCACC ACACAGCTGA ATAGTTTGGC TCACTGGATT TTGACCCTTT GTGCAATGGT TGAGGTAACC CGAGTTGACC CTGACATTGT GATGCAAGAA TCTGAGTTCT 5520**

**CCTTCTATGG TGATGACGAG GTGGTTTCGA CCAACCTCGA GTTGGATATG GTTAAGTACA CCATGGCTTT GAGGCGGTAC GGTCTCCTCC CGACTCGCGC GGACAAGGAG GAGGGACCTC TGGAGCGTCG CCAGACGCTG CAGGGCATCT CCTTCCTGCG CCGTGCGATA GTTGGTGACC AGTTTGGGTG GTACGGTCGT CTTGATCGTG CCAGCATCGA CCGCCAGCTC 5750**

**CTCTGGACTA AAGGACCTAA CCACCAGAAC CCCTTTGAGA CTCTCCCTGG ACATGCTCAG AGACCCTCCC AACTAATGGC CCTGCTCGGT GAGGCTGCCA TGCATGGTGA AAAGTATTAC AGGACTGTGG CTTCCCGTGT CTCCAAGGAG GCCGCCCAAA GTGGGATAGA AATGGTAGTC CCACGCCACC GATCTGTTCT GCGCTGGGTG CGCTTTGGAA CAATGGATGC 5980**

**TGAGACCCCG CAGGAACGCT CAGCAGTCTT TGTGAATGAG GATGAGTGAG GGCCCGTTTA AACCCGCTGA TCAGCCTCGA CTGTGCCTTC TAGTTGCCAG CCATCTGTTG TTTGCCCCTC CCCCGTGCCT TCCTTGACCC TGGAAGGTGC CACTCCCACT GTCCTTTCCT AATAAAATGA GGAAATTGCA TCGCATTGTC TGAGTAGGTG TCATTCTATT CTGGGGGGTG 6210**

**GGGTGGGGCA GGACAGCAAG GGGGAGGATT GGGAAGACAA TAGCAGGCAT GCTGGGGATG CGGTGGGCTC TATGGCTTCT GAGGCGGAAA GAACCAGCTG GGGCTCTAGG GGGTATCCCC ACGCGCCCTG TAGCGGCGCA TTAAGCGCGG CGGGTGTGGT GGTTACGCGC AGCGTGACCG CTACACTTGC CAGCGCCCTA GCGCCCGCTC CTTTCGCTTT CTTCCCTTCC 6440**

**TTTCTCGCCA CGTTCGCCGG CTTTCCCCGT CAAGCTCTAA ATCGGGGGCT CCCTTTAGGG TTCCGATTTA GTGCTTTACG GCACCTCGAC CCCAAAAAAC TTGATTAGGG TGATGGTTCA CGTAGTGGGC CATCGCCCTG ATAGACGGTT TTTCGCCCTT TGACGTTGGA GTCCACGTTC TTTAATAGTG GACTCTTGTT CCAAACTGGA ACAACACTCA ACCCTATCTC 6670**

**GGTCTATTCT TTTGATTTAT AAGGGATTTT GCCGATTTCG GCCTATTGGT TAAAAAATGA GCTGATTTAA CAAAAATTTA ACGCGAATTA ATTCTGTGGA ATGTGTGTCA GTTAGGGTGT GGAAAGTCCC CAGGCTCCCC AGCAGGCAGA AGTATGCAAA GCATGCATCT CAATTAGTCA GCAACCAGGT GTGGAAAGTC CCCAGGCTCC CCAGCAGGCA GAAGTATGCA 6900**

**AAGCATGCAT CTCAATTAGT CAGCAACCAT AGTCCCGCCC CTAACTCCGC CCATCCCGCC CCTAACTCCG CCCAGTTCCG CCCATTCTCC GCCCCATGGC TGACTAATTT TTTTTATTTA TGCAGAGGCC GAGGCCGCCT CTGCCTCTGA GCTATTCCAG AAGTAGTGAG GAGGCTTTTT TGGAGGCCTA GGCTTTTGCA AAAAGCTCCC GGGAGCTTGT ATATCCATTT 7130**

**TCGGATCTGA TCAGCACGTG TTGACAATTA ATCATCGGCA TAGTATATCG GCATAGTATA ATACGACAAG GTGAGGAACT AAACCATGGC CAAGTTGACC AGTGCCGTTC CGGTGCTCAC CGCGCGCGAC GTCGCCGGAG CGGTCGAGTT CTGGACCGAC CGGCTCGGGT TCTCCCGGGA CTTCGTGGAG GACGACTTCG CCGGTGTGGT CCGGGACGAC GTGACCCTGT 7360**

**TCATCAGCGC GGTCCAGGAC CAGGTGGTGC CGGACAACAC CCTGGCCTGG GTGTGGGTGC GCGGCCTGGA CGAGCTGTAC GCCGAGTGGT CGGAGGTCGT GTCCACGAAC TTCCGGGACG CCTCCGGGCC GGCCATGACC GAGATCGGCG AGCAGCCGTG GGGGCGGGAG TTCGCCCTGC GCGACCCGGC CGGCAACTGC GTGCACTTCG TGGCCGAGGA GCAGGACTGA 7590**

**CACGTGCTAC GAGATTTCGA TTCCACCGCC GCCTTCTATG AAAGGTTGGG CTTCGGAATC GTTTTCCGGG ACGCCGGCTG GATGATCCTC CAGCGCGGGG ATCTCATGCT GGAGTTCTTC GCCCACCCCA ACTTGTTTAT TGCAGCTTAT AATGGTTACA AATAAAGCAA TAGCATCACA AATTTCACAA ATAAAGCATT TTTTTCACTG CATTCTAGTT GTGGTTTGTC 7820**

**CAAACTCATC AATGTATCTT ATCATGTCTG TATACCGTCG ACCTCTAGCT AGAGCTTGGC GTAATCATGG TCATAGCTGT TTCCTGTGTG AAATTGTTAT CCGCTCACAA TTCCACACAA CATACGAGCC GGAAGCATAA AGTGTAAAGC CTGGGGTGCC TAATGAGTGA GCTAACTCAC ATTAATTGCG TTGCGCTCAC TGCCCGCTTT CCAGTCGGGA AACCTGTCGT 8050**

**GCCAGCTGCA TTAATGAATC GGCCAACGCG CGGGGAGAGG CGGTTTGCGT ATTGGGCGCT CTTCCGCTTC CTCGCTCACT GACTCGCTGC GCTCGGTCGT TCGGCTGCGG CGAGCGGTAT CAGCTCACTC AAAGGCGGTA ATACGGTTAT CCACAGAATC AGGGGATAAC GCAGGAAAGA ACATGTGAGC AAAAGGCCAG CAAAAGGCCA GGAACCGTAA AAAGGCCGCG 8280**

**TTGCTGGCGT TTTTCCATAG GCTCCGCCCC CCTGACGAGC ATCACAAAAA TCGACGCTCA AGTCAGAGGT GGCGAAACCC GACAGGACTA TAAAGATACC AGGCGTTTCC CCCTGGAAGC TCCCTCGTGC GCTCTCCTGT TCCGACCCTG CCGCTTACCG GATACCTGTC CGCCTTTCTC CCTTCGGGAA GCGTGGCGCT TTCTCATAGC TCACGCTGTA GGTATCTCAG 8510**

**TTCGGTGTAG GTCGTTCGCT CCAAGCTGGG CTGTGTGCAC GAACCCCCCG TTCAGCCCGA CCGCTGCGCC TTATCCGGTA ACTATCGTCT TGAGTCCAAC CCGGTAAGAC ACGACTTATC GCCACTGGCA GCAGCCACTG GTAACAGGAT TAGCAGAGCG AGGTATGTAG GCGGTGCTAC AGAGTTCTTG AAGTGGTGGC CTAACTACGG CTACACTAGA AGAACAGTAT 8740**

**TTGGTATCTG CGCTCTGCTG AAGCCAGTTA CCTTCGGAAA AAGAGTTGGT AGCTCTTGAT CCGGCAAACA AACCACCGCT GGTAGCGGTT TTTTTGTTTG CAAGCAGCAG ATTACGCGCA GAAAAAAAGG ATCTCAAGAA GATCCTTTGA TCTTTTCTAC GGGGTCTGAC GCTCAGTGGA ACGAAAACTC ACGTTAAGGG ATTTTGGTCA TGAGATTATC AAAAAGGATC 8970**

**TTCACCTAGA TCCTTTTAAA TTAAAAATGA AGTTTTAAAT CAATCTAAAG TATATATGAG TAAACTTGGT CTGACAGTTA CCAATGCTTA ATCAGTGAGG CACCTATCTC AGCGATCTGT CTATTTCGTT CATCCATAGT TGCCTGACTC CCCGTCGTGT AGATAACTAC GATACGGGAG GGCTTACCAT CTGGCCCCAG TGCTGCAATG ATACCGCGAG ACCCACGCTC 9200**

**ACCGGCTCCA GATTTATCAG CAATAAACCA GCCAGCCGGA AGGGCCGAGC GCAGAAGTGG TCCTGCAACT TTATCCGCCT CCATCCAGTC TATTAATTGT TGCCGGGAAG CTAGAGTAAG TAGTTCGCCA GTTAATAGTT TGCGCAACGT TGTTGCCATT GCTACAGGCA TCGTGGTGTC ACGCTCGTCG TTTGGTATGG CTTCATTCAG CTCCGGTTCC CAACGATCAA 9430**

**GGCGAGTTAC ATGATCCCCC ATGTTGTGCA AAAAAGCGGT TAGCTCCTTC GGTCCTCCGA TCGTTGTCAG AAGTAAGTTG GCCGCAGTGT TATCACTCAT GGTTATGGCA GCACTGCATA ATTCTCTTAC TGTCATGCCA TCCGTAAGAT GCTTTTCTGT GACTGGTGAG TACTCAACCA AGTCATTCTG AGAATAGTGT ATGCGGCGAC CGAGTTGCTC TTGCCCGGCG 9660**

**TCAATACGGG ATAATACCGC GCCACATAGC AGAACTTTAA AAGTGCTCAT CATTGGAAAA CGTTCTTCGG GGCGAAAACT CTCAAGGATC TTACCGCTGT TGAGATCCAG TTCGATGTAA CCCACTCGTG CACCCAACTG ATCTTCAGCA TCTTTTACTT TCACCAGCGT TTCTGGGTGA GCAAAAACAG GAAGGCAAAA TGCCGCAAAA AAGGGAATAA GGGCGACACG 9890**

**GAAATGTTGA ATACTCATAC TCTTCCTTTT TCAATATTAT TGAAGCATTT ATCAGGGTTA TTGTCTCATG AGCGGATACA TATTTGAATG TATTTAGAAA AATAAACAAA TAGGGGTTCC GCGCACATTT CCCCGAAAAG TGCCACCTGA CGTC 10044**
